# Outcomes of COVID-19 in kidney transplant recipients in the vaccination Era: A national multicenter cohort from Thailand

**DOI:** 10.1016/j.heliyon.2023.e22811

**Published:** 2023-11-24

**Authors:** Suwasin Udomkarnjananun, Stephen J. Kerr, Athiphat Banjongjit, Korntip Phonphok, Nuttasith Larpparisuth, Attapong Vongwiwatana, Kajohnsak Noppakun, Adisorn Lumpaopong, Thanom Supaporn, Cholatip Pongskul, Yingyos Avihingsanon, Natavudh Townamchai

**Affiliations:** a Division of Nephrology, Department of Medicine, Faculty of Medicine, Chulalongkorn University and King Chulalongkorn Memorial Hospital, Bangkok, Thailand; b Excellence Center for Organ Transplantation (ECOT), King Chulalongkorn Memorial Hospital, Thai Red Cross Society, Bangkok, Thailand; c Renal Immunology and Transplantation Research Unit, Faculty of Medicine, Chulalongkorn University, Bangkok, Thailand; d Biostatistics Excellence Center, Faculty of Medicine, Chulalongkorn University, Bangkok, Thailand; e Nephrology Unit, Vichaiyut Medical Center and Vichaiyut Hospital, Bangkok, Thailand; f Division of Nephrology, Department of Medicine, Faculty of Medicine, Rajavithi Hospital, Bangkok, Thailand; g Division of Nephrology, Department of Medicine, Faculty of Medicine, Siriraj Hospital, Mahidol University, Bangkok, Thailand; h Division of Nephrology, Department of Internal Medicine, Faculty of Medicine, Chiang Mai University, Chiang Mai, Thailand; i Division of Nephrology, Department of Pediatrics, Faculty of Medicine, Phramongkutklao Hospital and College of Medicine, Bangkok, Thailand; j Division of Nephrology, Department of Medicine, Faculty of Medicine, Phramongkutklao Hospital and College of Medicine, Bangkok, Thailand; k Division of Nephrology, Department of Medicine, Faculty of Medicine, Khon Kaen University, Khon Kaen, Thailand

**Keywords:** Bacterial infection, Booster vaccination, COVID-19, Kidney transplantation, Mortality, Pneumonia, Thailand

## Abstract

**Introduction:**

The mortality rate of coronavirus disease 2019 (COVID-19) in kidney transplant recipients (KTR) has significantly decreased with the implementation of vaccination programs. However, the real-world information on the impact of vaccinations, particularly in resource limited settings in Asia, is still limited.

**Methods:**

The Thai Transplant Society conducted a prospective multicenter cohort registry, including KTR diagnosed with COVID-19. Cox proportional hazards regression was used to examine factors associated with poor COVID-19 outcomes and complications, including death, COVID-19 pneumonia, and superimposed bacterial infection.

**Results:**

A total of 413 patients from 17 transplant centers who developed COVID-19 were analyzed. The COVID-19 mortality rate was 5.6 % and the incidence of pneumonia was 18.8 %. With each 10-year increase in age, the risk of death, pneumonia, and bacterial infection increased by 61 %, 32 %, and 43 %, respectively. A total of 11.4 % of KTR received one dose of COVID vaccination (incomplete vaccination), 25.7 % received two doses (complete primary vaccination), 42.6 % received three doses (first booster dose), and 10.4 % received four doses of vaccination (second booster dose). Even a single dose of vaccination significantly decreased the risk of death, pneumonia, and superimposed bacterial infection among KTR compared to those who remained unvaccinated. Completing the primary vaccination (2-dose) reduced the risk of death by 89 %, pneumonia by 88 %, and bacterial infection by 83 % compared to unvaccinated KTR. Receiving a booster dose (third or fourth dose) further reduced the risk of death by 94 %, pneumonia by 95 %, and bacterial infection by 96 % compared to unvaccinated individuals.

**Conclusions:**

This Asian cohort demonstrated that the mortality and complications of COVID-19 significantly decreased in KTR after the national immunization. Our study suggests that any type of COVID-19 vaccine can be beneficial in preventing adverse outcomes. Administering booster vaccinations is strongly recommended.

## 1 Introduction

Since the initial outbreak in December 2019, the coronavirus disease 2019 (COVID-19) has had a profound impact on kidney transplantation programs worldwide. In the case of kidney transplant recipients (KTR) who are routinely administered immunosuppressive medications, the rates of mortality and morbidity associated with COVID-19 have been notably elevated compared to the general population [1]. Throughout the initial year of the pandemic in 2021, prior to the availability of a vaccine, several factors were identified as being associated with increased mortality among KTR. These factors included the utilization of immunosuppressants, advanced age, deceased donor kidney transplantation, diabetes mellitus, preexisting cardiovascular diseases, and active malignancies [2].

Thailand was the first country outside of China to have a confirmed case of COVID-19 [3]. After experiencing the first COVID-19 wave when vaccine availability in Thailand was limited, there was a large number of fatalities among KTR, resulting in the Thai Transplant Society establishing the Thai COVID-19 KTR registry. The primary objective of this registry was to facilitate a multicenter cohort study that would gather comprehensive clinical data and outcomes of Thai KTR who became infected with SARS-CoV-2. The cohort was launched in March 2021, coinciding with the introduction of the first national SARS-CoV-2 vaccine in Thailand.

At present, most of the information about COVID-19 outcomes in KTR comes from Western countries [[4], [5], [6], [7], [8], [9], [10], [11], [12], [13], [14]]. This leaves a notable gap in knowledge about Asian KTR, where a wider variety of vaccine types, including inactivated, viral vector, and mRNA vaccines, have been used. In this study, we present data from the Thai COVID-19 KTR registry, encompassing COVID-19 outcomes and the identification of factors associated with unfavorable outcome.

## 2 Methods

### 2.1 Study designs and study population

This multicenter prospective cohort study was initiated on March 1, 2021. Invitations for participation were sent to all transplant centers in Thailand, and an online case record form was distributed to transplant coordinators. Adult (>18-year-old) KTR with confirmed COVID-19, determined through either antigen test kit (ATK) or polymerase chain reaction (PCR) of nasopharyngeal or oropharyngeal swab samples, were included in the cohort. Demographic and transplantation information, including immunosuppressive medications and SARS-CoV-2 vaccination history, were recorded. The first vaccine deployment in Thailand took place on February 28, 2021, when the ChAdOx1 nCoV-19 (Oxford-AstraZeneca COVID-19 vaccine) and the inactivated vaccine (Sinovac COVID-19 vaccine) were simultaneously launched. This presented an opportunity for our cohort to observe differences in COVID-19 outcomes among KTR during the ongoing vaccination period, until recommendations were made for booster doses, when mRNA vaccines were more readily available. The transplant coordinators in each participating center were responsible for reporting the information of KTR when they were diagnosed with COVID-19. This information included details about the type and number of vaccinations, and it was conducted as a standard protocol established by the Thai Transplant Society. The cohort concluded in October 2022, when Thailand's Center of COVID-19 Situation Administration (CCSA) reclassified COVID-19 from a dangerous communicable disease to a communicable disease under surveillance, thereby officially ending the nationwide COVID-19 emergency regulations.

### 2.2 Outcome measurement

The primary outcome of this study was COVID-19-related mortality in KTR. The study also investigated the clinical manifestations and complications of COVID-19 as secondary outcomes. Clinical manifestations of COVID-19 were categorized into four main group: asymptomatic, symptomatic without pneumonia, mild-to-moderate pneumonia (defined by new opacity or infiltration on chest x-ray or computed tomography (CT) scan, without an alternative explanation for the findings), and severe pneumonia (defined by the aforementioned new infiltration and an SpO_2_ ≤93 % or requirement of mechanical ventilation). The duration of symptoms was defined as the period between symptom onset and the resolution of primary symptoms if not admitted, or the period between symptom onset and hospital discharge if admitted. For asymptomatic cases, the duration of symptoms was defined as zero. The incidence of superimposed bacterial infection (defined by the positive bacterial culture from blood, sputum, urine, or other body fluids) was documented and analyzed as a complication of interest in this study. Information about COVID-19 outcomes was reported by the transplant coordinators at each transplant center through an online case record form. This information was subsequently reviewed and verified by the investigators involved in this study.

### 2.3 Statistical analysis

Continuous data are presented as mean ± standard deviation (SD) or median with interquartile range (IQR). Categorical data are reported as numbers with percentages. Cox proportional hazards regression was used to analyze the hazard ratio (HR) for factors associated with death, pneumonia, and superimposed bacterial infection in KTR with COVID-19. Variables with a p-value ≤0.2 from the univariable analysis were included in the multivariable models. To minimize immortal time bias, we modelled COVID-19 successive vaccinations received as time updated variables for each KTR, to accurately attribute time at risk after different numbers of vaccinations. Recipients were considered vaccinated ≥14 days after receiving a specific vaccine dose. Curves of the survivor function were created from multivariable Cox proportional hazards regression, adjusted for covariates, to demonstrate associations between the number of vaccinations and death, pneumonia, or superimposed bacterial infection in KTR. Lastly, given the very strong protection against outcomes in those who received at least 2 vaccines, we assessed the impact of viral vector and mRNA vaccines versus whole cell vaccines as a reference, adjusting for the covariates that were used in the multivariable model of vaccine numbers. Non-COVID-related deaths did not occur among the included recipients in our cohort, and death was not considered a competing event in any time to event models since the incidence of death was low, and the majority of patients who died developed superimposed bacterial infection or pneumonia before they died, so the cumulative incidence of these other events was not impacted., Univariable and multivariable linear regression were performed to explore associations between potential predictor variables and the duration of COVID-19 symptoms. All statistical analyses were conducted using Stata 17.0 (StataCorp LLC, College Station, TX).

### 2.4 Ethical considerations

This study was approved by the Institutional Review Board of the Faculty of Medicine, Chulalongkorn University, Bangkok, Thailand (IRB No. 409/66). Informed consent was obtained from all subjects and/or their legal guardian(s).

## 3 Results

### 3.1 Baseline characteristics of the included KTR and clinical outcomes of COVID-19

**Fig. 1:**
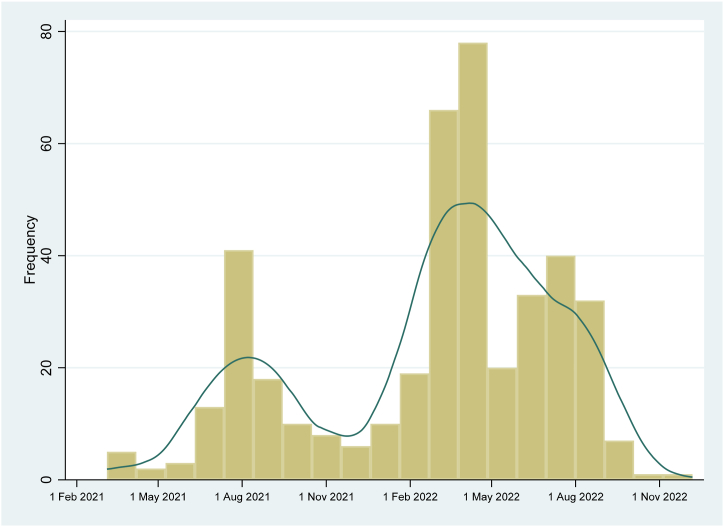
Histogram and Kernel density estimate of number of kidney transplant recipients with COVID-19 after vaccines were distributed in Thailand (March 1, 2021 as the starting date).

A total of 413 KTR diagnosed with COVID-19 from 17 transplant centers throughout Thailand were included in this multicenter cohort study. Since the cohort was established during a period of intense COVID-19 testing and surveillance, rigorous data collection protocols were implemented and data submitted by participating transplant centers were comprehensive, with no missing outcomes of interest among the included KTR. Table 1 presents the clinical characteristics and vaccination history of the participants at the time of COVID-19 diagnosis. The majority of the included KTR were female (56.9 %) with an average age of 47.0 ± 11.4 years. The median duration from kidney transplantation to COVID-19 diagnosis was 5.1 (2.4–8.8) years. Most KTR had received 3 doses of vaccine (42.6 %) at the time of diagnosis, primarily consisting of 2 doses of viral vector vaccine (ChAdOx1 nCoV-19) followed by an mRNA vaccine (either BNT162b2 Pfizer vaccine or mRNA-1273 Moderna vaccine). The immunosuppressive medications received by the KTR included tacrolimus (83.3 %), mycophenolic acid (MPA) (85.0 %), and prednisolone (69.7 %). Fig. 1 illustrates the distribution of the number of KTR diagnosed with COVID-19 between March 1, 2021 and October 1, 2022. The number of cases peaked in August 2021 and April 2022, coinciding with waves of the delta and omicron variants of concerns (VOCs) in Thailand, respectively [15].

**Table 1:** Demographic information of kidney transplant recipient at the time of COVID-19 infection.

Variables	Values
N	413
Age, years (mean ± SD)	47.0 ± 11.4
Female, n (%)	235 (56.9 %)
Diabetes mellitus, n (%)	81 (19.6 %)
Body mass index, kg/m^2^ (mean ± SD)	23.5 ± 4.7
Living donor kidney transplantation, n (%)	138 (33.4)
HLA mismatch (mean ± SD)	2.9 ± 2.0
PRA (median and IQR)	0 (0–5)
Multiorgan transplantation, n (%)	12 (2.9 %)
Immunosuppressive medication, n (%)	
Tacrolimus	344 (83.3 %)
Cyclosporine	36 (8.7 %)
Mycophenolic acid	351 (85.0 %)
Mammalian target of rapamycin inhibitor	60 (14.5 %)
Prednisolone	288 (69.7 %)
Azathioprine/leflunomide	13 (3.2 %)
Time after kidney transplantation, years (median and IQR)	5.1 (2.4–8.8)
Serum creatinine at diagnosis, mg/dL (median and IQR)	1.47 (1.15–2.16)
Vaccination history when COVID-19 was diagnosed, n (%)	
No vaccination	41 (9.9 %)
1 dose of vaccine (incomplete vaccination)	47 (11.4 %)
2 doses of vaccine	106 (25.7 %)
3 doses of vaccine	176 (42.6 %)
4 doses of vaccine	43 (10.4 %)
Type of the first 2-dose vaccination received (n = 325), n (%)	
Inactivated vaccine	58 (17.8 %)
Viral vector vaccine	232 (71.4 %)
mRNA vaccine	35 (10.8 %)
Type of the third (booster) vaccine received (n = 219), n (%)	
Viral vector vaccine	32 (14.6 %)
mRNA vaccine	187 (85.4 %)
Type of the fourth (booster) vaccine received (n = 43), n (%)	
mRNA vaccine	43 (100 %)

HLA; human leukocyte antigen, PRA; panel reactive antibody.

Table 2 presents the clinical outcomes and complications of COVID-19 in the KTR population. The majority of KTR exhibited symptomatic infection without pneumonia (72.2 %), with predominant symptoms being upper respiratory tract infection symptoms such as rhinorrhea, sore throat, and cough. Pneumonia was observed in 18.8 % of COVID-19-infected KTR. The total duration of symptoms was significantly longer in KTR with pneumonia compared to those without pneumonia (16 (12–26) days vs. 7 (4–10) days; p-value <0.001). Of the total 413 KTR with COVID-19, 36 (8.7 %) developed superimposed bacterial infection and 23 (5.6 %) died.

**Table 2:** Outcomes and complications of COVID-19 infection.

Outcomes and complications	Values
Clinical manifestation, n (%)	
Asymptomatic	37 (9.0 %)
Symptomatic without pneumonia	298 (72.2 %)
Mild-to-moderate pneumonia (SpO_2_ >93 %)	41 (9.9 %)
Severe Pneumonia (SpO_2_ ≤93 % or required mechanical ventilation)	37 (8.9 %)
Duration of symptoms, days (median and IQR)	8 (5–11)
Symptomatic without pneumonia	8 (5–10)
Mild-to-moderate pneumonia (SpO_2_ >93 %)	14 (10–16)
Severe Pneumonia (SpO_2_ ≤93 % or required mechanical ventilation)	25 (15–30)
Superimposed bacterial infection, n (%)	36 (8.7 %)
Death, n (%)	23 (5.6 %)

### 3.2 Factors associated with poor outcomes of COVID-19

**Fig. 2:**
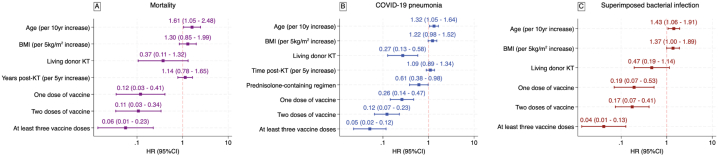
Hazard ratio from multivariable Cox proportional hazards regression for (A) death, (B) COVID-19 pneumonia, and (C) superimposed bacterial infection in kidney transplant recipients with COVID-19.

Clinical variables at the time of COVID-19 diagnosis were analyzed to assess their association with poor outcomes, including death, pneumonia, and superimposed bacterial infection. The results of univariable and multivariable Cox regression for poor outcomes are presented in Supplementary Tables S1–S3, displaying the HR and 95%CI for each variable. The adjusted hazard ratios for each outcome are succinctly presented in Fig. 2A for death, Fig. 2B for COVID-19 pneumonia, and Fig. 2C for superimposed bacterial infection. Advancing age exhibited an association with all adverse outcomes. Notably, recipients of living donor kidney transplantation and those on immunosuppression regimens containing prednisolone were associated with a reduced risk of COVID-19 pneumonia. Additionally, body mass index (BMI) demonstrated an association with an elevated risk of superimposed bacterial infection. Across all outcomes, individuals who received COVID-19 vaccination were consistently associated with a decreased risk of adverse outcomes, as elaborated below.

**Fig. 3:**
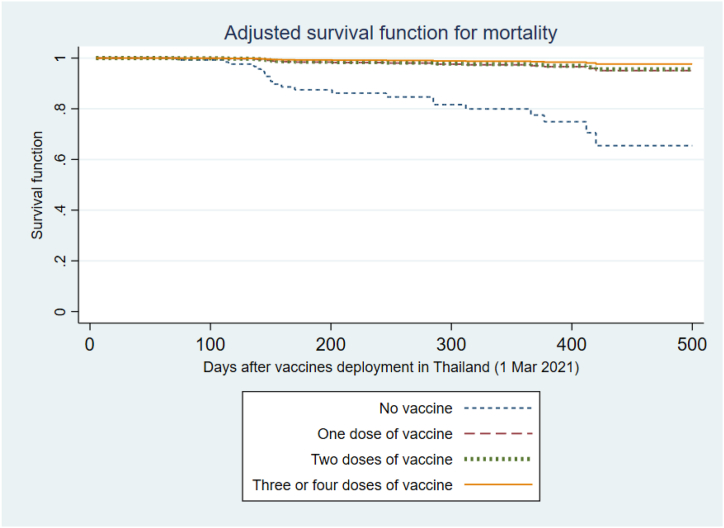
Adjusted function for risk of death among kidney transplant recipients with COVID-19 following vaccine deployments in Thailand, categorized based on the number of vaccinations received. The model was adjusted for age, BMI, living donor kidney transplantation, and duration after transplantation.

After adjusting for BMI, living donor transplant and years post KT, older age demonstrated a significant association with increased risk of death (HR 1.61 per 5-year increase; 95%CI 1.05–2.48; p-value 0.03). Recipients who received at least one vaccination had significant reductions in the risk of death compared to those who had not been vaccinated (HR 0.12; 95%CI 0.03–0.41; for 1-dose of vaccine; HR 0.11; 95%CI 0.03–0.34 for 2 vaccine doses, and HR 0.06; 95%CI 0.01–0.23; for recipients of at least 3-doses of vaccine; P for trend <0.0001). The adjusted mortality survivor functions are presented in Fig. 3.

**Fig. 4:**
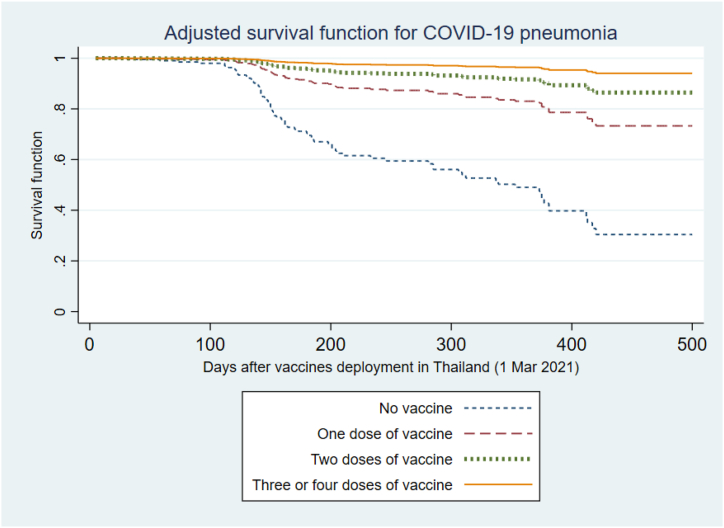
Adjusted survival function for risk of pneumonia among kidney transplant recipients with COVID-19 following vaccine deployments in Thailand, categorized based on the number of vaccinations received. The model was adjusted for age, BMI, living donor kidney transplantation, prednisolone-containing regimen, and duration after transplantation.

After adjusting for BMI and years post-KT, COVID-19 pneumonia was also associated with increasing age (HR 1.32; 95%CI 1.05–1.64 per 10-year increase, p-value 0.016), while living donor transplantation (HR 0.27; 95%CI 0.13–0.58; p-value 0.001) and being treated with prednisolone-containing immunosuppressive regimen decreased the risk (HR 0.61; 95%CI 0.38–0.98; p-value 0.041). The risk of pneumonia was significantly decreased with increasing numbers of vaccine doses: 1 dose (HR 0.26; 95%CI 0.14–0.47), 2 doses (HR 0.12; 95%CI 0.07–0.23), and at least 3 doses of the vaccine (HR 0.05; 95%CI 0.02–0.12) compared to unvaccinated KTR (p-value for trend <0.0001). Adjusted pneumonia survivor functions are presented in Fig. 4.

**Fig. 5:**
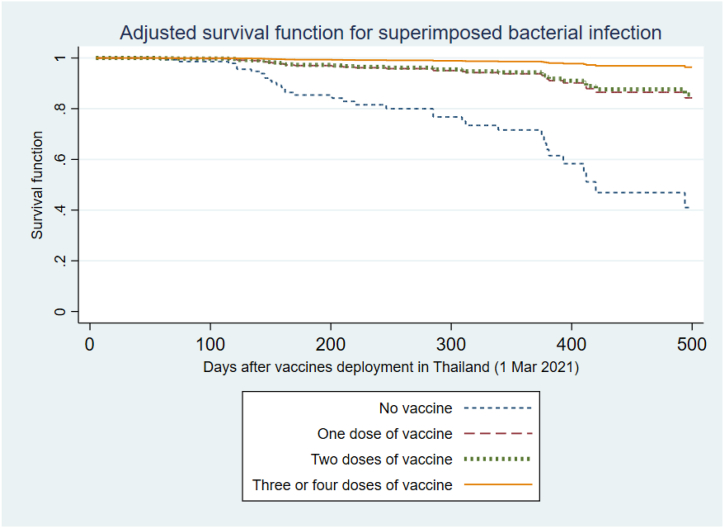
Adjusted survival function for risk of superimposed bacterial infection among kidney transplant recipients with COVID-19 following vaccine deployments in Thailand, categorized based on the number of vaccinations received. The model was adjusted for age, BMI, and living donor kidney transplantation.

In a multivariable model adjusting for BMI and whether the transplant was from a living donor, the risk of superimposed bacterial infection also increased with age (HR 1.43 per 10-year increase; 95%CI 1.06–1.91; p-value 0.017), and BMI (HR 1.37 per 5kg/m2 increase (95%CI 1.0–1.89; p-value 0.05). The risk decreased with increasing vaccine doses (HR 0.19; 95%CI 0.07–0.53 for 1-dose, HR 0.17; 95%CI 0.07–0.41 for 2-doses, and HR 0.04; 95%CI 0.01–0.13 for at least 3-doses (p-value for trend <0.0001). The adjusted survival curve is demonstrated in Fig. 5.

Factors associated with duration of COVID-19 symptoms were analyzed using univariable and multivariable linear regression (Table 3). The multivariable analysis revealed at the time of COVID-19 diagnosis, high body mass index (BMI) significantly increased the duration of symptoms (β-coefficient 0.20; 95%CI 0.01 to 0.40; p-value 0.044). In contrast, living donor transplantation, receipt of 2 doses of vaccine, and receipt of at least 3 doses of vaccine were associated with a decreased duration of symptoms (β-coefficient −2.27; 95%CI -4.32 to −0.22; p-value 0.03, β-coefficient −4.67; 95%CI -8.17 to −1.17; p-value 0.009, and β-coefficient −6.82; 95%CI -10.95 to −3.56; p-value <0.001, respectively).

**Table 3:** Univariable and multivariable linear regression for duration of COVID-19 symptoms.

Variables	Univariable analysis			Multivariable analysis		
	β-coefficient	95%CI	p-value	β-coefficient	95%CI	p-value
Age (per 10 years increased)	0.90	0.03 to 1.77	0.043	0.76	−0.09 to 1.61	0.08
Female	0.93	−1.08 to 2.94	0.36	–	–	–
Diabetes mellitus	1.42	−1.09 to 3.92	0.27	–	–	–
BMI (per 1 kg/m^2^ increased)	0.26	0.05 to 0.47	0.016	0.20	0.01 to 0.40	0.044
Living donor transplantation	−3.41	−5.50 to −1.32	0.001	−2.27	−4.32 to −0.22	0.03
HLA mismatch (per 1 mismatch increased)	0.14	−0.36 to 0.64	0.58	–	–	–
CNI with MPA regimen (compared with other regimens)	0.85	−1.61 to 3.32	0.50	–	–	–
Prednisolone-containing regimen	−3.37	−5.52 to −1.22	0.002	−1.72	−3.82 to 0.39	0.11
Serum creatinine (per 1 mg/dL increased)	0.37	−0.05 to 0.79	0.09	0.38	−0.01 to 0.77	0.054
Time after transplantation (per 1 year increased)	0.15	−0.01 to 0.32	0.07	0.11	−0.07 to 0.29	0.25
One dose of vaccine received (compared with no vaccination)	2.55	−1.51 to 6.61	0.22	–	–	–
Two doses of vaccine received (compared with no vaccination)	−5.52	−9.01 to −2.03	0.02	−4.67	−8.17 to −1.17	0.009
At least three doses of vaccine received (compared with no vaccination)	−7.86	−11.10 to −4.63	<0.001	−6.82	−10.95 to −3.56	<0.001

BMI; body mass index, CNI; calcineurin inhibitor, HLA; human leukocyte antigen, MPA; mycophenolic acid.

Exploratory analyses were conducted to understand associations between the type of vaccination and outcomes. Among the 106 KTR who completed the 2-dose “primary” vaccination and had no booster doses, the distribution of vaccine types was as follows: viral vector vaccine 56 (52.8 %), inactivated vaccine 15 (14.2 %), mRNA vaccine 15 (14.2 %), and heterologous vaccine 20 (18.9 %). Viral vector vaccine (aHR 0.24; 95%CI 0.02–2.73; p-value = 0.24) and mRNA vaccine (aHR 0.91; 95%CI 0.07–10.50; p-value 0.94) did not significantly reduced mortality compared with inactivated vaccine. Both viral vector vaccine and mRNA vaccines were associated with a reduced relative risk of COVID-19 pneumonia compared to inactivated vaccine (aHR 0.15; 95%CI 0.05–0.47; p-value = 0.001 and aHR 0.23; 95%CI 0.06–0.93; p-value 0.04). Lastly, no significant differences were found in superimposed bacterial infection for viral vector vaccine (aHR 0.68; 95%CI 0.08–5.85; p-value 0.73) or mRNA vaccine (aHR 1.27; 95%CI 0.12–12.56; p-value 0.84) when compared with inactivated vaccine.

## 4 Discussions

The present study provides an overview of outcomes in a cohort of Thai KTR who were infected with SARS-CoV-2 infection. This cohort represents the first group of COVID-19 patients observed in South East Asia during the vaccination period. The mortality rate within our cohort was 5.6 %, with approximately one-fifth of all KTR cases developing pneumonia. Age was identified as a significant factor contributing to mortality, pneumonia, and superimposed bacterial infection. Being a recipient of deceased donor kidney transplantation increased the risk of COVID-19 pneumonia. Furthermore, deceased donor kidney transplantation status was also associated with an extended duration of COVID-19 symptoms, similar to BMI. Our findings indicate that even a single dose of vaccination significantly decreased the risk of death, pneumonia, and superimposed bacterial infection among KTR compared to those who remained unvaccinated. There was an incremental protective benefit of vaccination against these outcomes with each additional vaccination received. Furthermore, booster doses provide a significant additive benefit beyond the standard primary two-dose vaccination regimen. These results underscore the importance of multiple vaccinations and booster doses in maximizing the effectiveness of vaccination in KTR.

Prior to the pandemic, Thailand boasted an advanced kidney transplant program within Asia, encompassing ABO- and human leukocyte antigen (HLA)-incompatible transplantations, as well as transplants performed on HIV-positive recipients [[16], [17], [18], [19]]. However, COVID-19 substantially impacted all kidney transplant activities in the country due to overwhelmed hospital resources and travel restrictions imposed between provinces. Since the implementation of the national COVID-19 vaccination program, transplant activities gradually resumed and are now at pre-pandemic levels.

The results of this study demonstrated that age was an independent factor that contributes to poor outcomes in COVID-19 among KTR. With every 10-year increase in age, the adjusted relative mortality rate of COVID-19 increased by 61 %, the adjusted risk of pneumonia increased by 32 %, and the adjusted risk of superimposed bacterial infection increased by 43 %. These findings align with previous meta-analyses and studies conducted on the general population and KTR, which have shown a linear relationship between age and the risk of hospitalization and death from COVID-19 [2,20,21]. Immunosenescence is associated with increased susceptibility to SARS-CoV-2 infection and reduced viral clearance capability [21,22]. Age-related degeneration or remodeling of organs also lowers the cardio-respiratory reserve in the elderly, putting them at higher risk for worse outcomes compared to younger patients [21]. SARS-CoV-2 enters human cells through the angiotensin converting enzyme 2 (ACE2) receptor, which is known to increase with age [22,23]. It is hypothesized that the increased presence of ACE2 receptor in many organs in the elderly may promote viral entry and lead to poor outcomes [22].

In our study, increased BMI was associated with a prolonged duration of COVID-19 symptoms. This finding could be explained by the effects of obesity and adipose tissue on dysregulation of adaptive and innate immunity, chronic inflammation, and gut dysbiosis, which contribute to a higher rate of complications [24]. Deceased donor kidney transplantation was associated with and increased risk of COVID-19 pneumonia and prolonged symptom duration. This may be attributed to inferior allograft function resulting from perioperative ischemic reperfusion injury and a higher degree of net immunosuppression compared to living donor kidney transplantation [17,25]. Interestingly, prednisolone-containing regimen was associated with reduced risk of COVID-19 pneumonia. Steroids have shown effects on COVID-19 severity at various disease stages. Early administration of inhaled budesonide reduced the risk of serious COVID-19 outcomes and shortened recovery time [26]. A large randomized controlled trial demonstrated that corticosteroids reduced mortality in patients receiving invasive mechanical ventilation or oxygen therapy [27]. These beneficial effects are attributed to the pleiotropic actions of corticosteroids, which include the inhibition of pro-inflammatory cytokines and proteins, suppression of chemokines and adhesion molecules, induction of anti-inflammatory cytokines and proteins, and suppression of leukocyte maturation, differentiation, and proliferation [28]. These mechanisms might result in a reduced severity of COVID-19 among KTR who were receiving prednisolone-containing regimen. However, it is important to interpret our results cautiously, as information regarding treatment interventions, including corticosteroid administration or withdrawal during COVID-19 was not available in the cohort.

Initially, the mortality rate of COVID-19 in KTR was as high as 30 %, but it has decreased to less than 10 % in subsequent periods [2,14,29,30]. Several factors have contributed to this improvement, including better knowledge of patient care, the use of corticosteroids, appropriate utilization of antiviral medications and monoclonal antibodies, and non-pharmacologic interventions [[31], [32], [33], [34], [35]]. However, largest public health benefits were due to vaccination programs. Previous studies have shown that KTR have an inferior immune response to SARS-CoV-2 vaccination compared to the general population, and booster doses are necessary to enhance immune response for protection against SARS-CoV-2 infection and its severe complications [4,11]. However, the real-world efficacy of booster vaccinations in KTR, especially in Eastern countries with differing vaccine distribution and socioeconomic factors from Western countries, still require more data [9,[11], [12], [13], [14],^36^].

The results of this study provide clear evidence that a complete primary series (2-dose) of COVID-19 vaccination reduces mortality in KTR, and the administration of booster doses further decreases the risk of death, COVID-19 pneumonia, and superimposed bacterial infection. These findings confirm that despite the inferior humoral and cellular immune response observed in KTR following SARS-CoV-2 vaccination compared to non-transplant population [4,5,7,37], there is a definitive benefit from vaccination, and the administration of third or fourth booster doses is strongly recommended. The cellular immune response plays a major role in protecting against disease development and reducing the severity of COVID-19, while the humoral response is responsible for infectivity and transmission [38]. It is important to note that our study focused on the outcomes of KTR who were already infected with SARS-CoV-2, rather than the infection rate.

Inactivated SARS-CoV-2 vaccine has been used mostly in the Eastern countries and only few studies have addressed the comparative analysis of immune response between inactivated vaccines versus others. An experimental study demonstrated that the inactivated vaccine induced lower magnitude of SARS-CoV-2 spike-specific T cells than the mRNA vaccine, but the overall T cell responses were comparable between the two. Additionally, inactivated vaccine selectively induced CD4 T cells, unlike mRNA vaccine that stimulated both CD4 and CD8 T cells [39]. Another study of patient with multiple sclerosis showed that the serologic response from inactivated vaccine was lower than from the mRNA SARS-CoV-2 vaccine [40]. Our study has an opportunity to examine the potential difference among different type of vaccinations, although the number of who receiving inactivated and mRNA vaccines versus the viral vector vaccine, without receiving booster immunization was small. Viral vector and mRNA vaccine were not significantly different in reducing the HR of death and superimposed bacterial infection in KTR who competed primary 2-dose vaccination, compared with an inactivated vaccine, but as these complications were infrequent, our analyses had limited power to assess relative effects. However, both viral vector and mRNA significantly decreased the HR of COVID-19 pneumonia compared with inactivated vaccine. This finding might relate to the above-mentioned different immunological aspects between inactivated vaccine and other vaccines. Data from other countries that utilized the inactivated vaccine in KTR may yield additional insights regarding the efficacy of specific primary vaccination schedules [41].

Since the poor COVID-19 outcomes rarely occurred in KTR who received a booster dose, we did not explore the efficacy of each primary-booster combination. The appropriate interval between each COVID-19 booster dose remains question, as immunity against SARS-CoV-2 diminishes over time [42]. Ideally, the booster dose should provide durable protection, involving both cellular and humoral immunity, be affordable, and have a less frequent administration interval than or equal to annually. The primary focus should be on preventing severe disease, hospitalization, and death, rather than solely targeting transmission or infection rates [38].

There are several notable strengths in this study. Firstly, it is the first to report outcomes of KTR with COVID-19 in Asia during the period of primary and booster vaccinations, providing valuable insights into a region with a different COVID-19 vaccine distribution compared to Western countries. Secondly, the study identified the factors associated with death, COVID-19 pneumonia, superimposed bacterial infection, and prolonged duration of COVID-19 symptoms in KTR. The study highlights the effects of booster doses in this population. However, there are limitations to consider. Firstly, the sample size in this study is relatively small compared to other registries. Nevertheless, data were collected from 17 transplant centers covering all regions in Thailand, which enhances the generalizability of the results. Secondly, information on specific SARS-CoV-2 strains and VOCs could not be captured, as this data was only available in limited tertiary hospitals and most KTR did not undergo testing for VOCs. Thirdly, while the study focused on clinical endpoints, it did not investigate humoral and cellular immunity at the time of COVID-19 diagnosis or following vaccinations, measured by the anti-spike antibody levels or enzyme-linked immunosorbent spot (ELISPOT) assay, both of which are inferior in KTR than in dialysis or the general population [4,37]. Fourthly, KTR without COVID-19 were not included in the study as the primary objective was to examine factors associated with disease severity among those with SARS-CoV-2 infection. Therefore, comparisons between infected and non-infected KTR were beyond the scope of this study. Finally, we acknowledge that there is a possibility that some kidney transplant recipients with COVID-19 may not have been included in our study, as our data relied on the information provided by the participating transplant centers. However, despite this potential limitation, we believe that our multicenter cohort can still serve as representative data for the Thai kidney transplant population.

In conclusion, this multicenter cohort study of KTR with COVID-19 demonstrated that elderly, deceased donor kidney transplantation, and higher BMI were associated with poor outcomes. KTR with these risks should be strongly encouraged and prioritized for SARS-CoV-2 vaccinations, particularly the booster doses. Completion of the primary COVID-19 vaccination series provided protection against death, COVID-19 pneumonia, and superimposed bacterial infection. Booster doses further reduced the risk of these outcomes, emphasizing the importance of COVID-19 vaccine booster in KTR to mitigate the severity of COVID-19.

## Ethical statement

This study was approved by the Institutional Review Board of the Faculty of Medicine, Chulalongkorn University, Bangkok, Thailand (IRB No. 409/66). It was conducted in compliance with the international guidelines for human research protection as described in the Declaration of Helsinki, The Belmont Report, CIOMS Guideline and International Conference on Harmonization in Good Clinical Practice (ICH-GCP).

## Data availability statement

All data relevant to this study has been included in the manuscript. No data associated with this study been deposited in a publicly available repository. The data code supporting the findings of this study are available from the corresponding author upon reasonable request.

## Authorship

S.U. involved with the first draft of the manuscript, study design, statistical analyses, data collection, and funding acquisition. S.K. involved with the statistical analysis and manuscript review. A.B. involved with data collection and study design. K.P., N.L., A.V., K.N., A.L., T.S., C.P., and Y.A. involved with data collection and manuscript review. N.T. involved with study design, data collection, and manuscript review.

## FUNDING

This study is supported by Ratchadapisek Sompoch Endowment Fund of 10.13039/501100002873Chulalongkorn University (CU_GR_63_157_30_60).

## Key learning points

### What was known

• Coronavirus disease 2019 (COVID-19) has resulted in significant morbidity and mortality among kidney transplant recipients (KTR).
• While numerous studies have demonstrated the protective efficacy of COVID-19 vaccines, there is a lack of real-world data specifically focusing on KTR, especially in the context of Asian countries that utilize a combination of inactivated, viral vector, and mRNA vaccines.

### This study adds

• This study presents the real-world efficacy of COVID-19 vaccines based on data from a national registry of KTR in Thailand, which covers from the initial phase of primary vaccinations to booster vaccinations.
• Older age, recipient of a deceased donor kidney transplantation, and higher body mass index were associated with poor COVID-19 outcomes.
• Even a single dose of vaccination against COVID-19 was shown to reduce the risk of death, COVID-19 pneumonia, and superimposed bacterial infection in KTR. Receiving booster doses further contributed to reducing the risk of adverse outcomes among KTR.

### Potential impact

• The advantages of COVID-19 vaccination in Eastern countries, where inactivated, viral vector, and mRNA vaccines are employed, correspond closely to those observed in Western countries.
• Despite immunological investigations highlighting a comparatively weaker immune response to COVID-19 vaccination among transplant recipients in comparison to the general population, administration of a single vaccine dose still holds the potential to mitigate adverse COVID-19 outcomes.
• The efficacy of booster doses can be translated into real-world improvements in clinical outcomes, which strongly supports the recommendation of extended primary series vaccination.

## CRediT authorship contribution statement

**Suwasin Udomkarnjananun:** Writing – review & editing, Writing – original draft, Methodology, Investigation, Formal analysis, Data curation, Conceptualization. **Stephen J. Kerr:** Writing – review & editing, Formal analysis. **Athiphat Banjongjit:** Writing – review & editing, Data curation. **Korntip Phonphok:** Writing – review & editing. **Nuttasith Larpparisuth:** Writing – review & editing. **Attapong Vongwiwatana:** Writing – review & editing. **Kajohnsak Noppakun:** Writing – review & editing. **Adisorn Lumpaopong:** Writing – review & editing. **Thanom Supaporn:** Writing – review & editing. **Cholatip Pongskul:** Writing – review & editing. **Yingyos Avihingsanon:** Writing – review & editing. **Natavudh Townamchai:** Writing – review & editing, Supervision.

## Declaration of competing interest

The authors declare that they have no known competing financial interests or personal relationships that could have appeared to influence the work reported in this paper.
