# Supplementary material for: Outcomes of COVID-19 in kidney transplant recipients in the vaccination Era: A national multicenter cohort from Thailand
Source: Heliyon. 2023 Nov 24;9(12):e22811. doi: 10.1016/j.heliyon.2023.e22811 (PMC10711172; doi:10.1016/j.heliyon.2023.e22811)
Supplement: Multimedia component 1 [file mmc1.docx]

**Outcomes of COVID-19 in Kidney Transplant Recipients in the Vaccination Era: A National Multicenter Cohort from Thailand**

Suwasin Udomkarnjananun^1,2,3^, MD, PhD, Stephen J Kerr^4^, PhD, Athiphat Banjongjit^1,5^, MD, Korntip Phonphok^6^, MD, Nuttasith Larpparisuth^7^, MD, Attapong Vongwiwatana^7^, MD, Kajohnsak Noppakun^8^, MD, Adisorn Lumpaopong^9^, MD, Thanom Supaporn^10^, MD, Cholatip Pongskul^11^, MD, Yingyos Avihingsanon^1,2,3^, MD, Natavudh Townamchai^1,2,3^, MD

^1^Division of Nephrology, Department of Medicine, Faculty of Medicine, Chulalongkorn University and King Chulalongkorn Memorial Hospital, Bangkok, Thailand

^2^Excellence Center for Organ Transplantation (ECOT), King Chulalongkorn Memorial Hospital, Thai Red Cross Society, Bangkok, Thailand.

^3^Renal Immunology and Transplantation Research Unit, Faculty of Medicine, Chulalongkorn University, Bangkok, Thailand.

^4^Biostatistics Excellence Center, Faculty of Medicine, Chulalongkorn University, Bangkok, Thailand

^5^Nephrology Unit, Vichaiyut Medical Center and Vichaiyut Hospital, Bangkok, Thailand

^6^Division of Nephrology, Department of Medicine, Faculty of Medicine, Rajavithi Hospital, Bangkok, Thailand

^7^Division of Nephrology, Department of Medicine, Faculty of Medicine, Siriraj Hospital, Mahidol University, Bangkok, Thailand

^8^Division of Nephrology, Department of Internal Medicine, Faculty of Medicine, Chiang Mai University, Chiang Mai, Thailand

^9^Division of Nephrology, Department of Pediatrics, Faculty of Medicine, Phramongkutklao Hospital and College of Medicine, Bangkok, Thailand

^10^Division of Nephrology, Department of Medicine, Faculty of Medicine, Phramongkutklao Hospital and College of Medicine, Bangkok, Thailand

^11^Division of Nephrology, Department of Medicine, Faculty of Medicine, Khon Kaen University, Khon Kaen, Thailand

**Supplementary Table S1**: Univariable and multivariable Cox proportional hazards regression for death in kidney transplant recipients with COVID-19 infection.

| **Variables** | **Univariable analysis** | | | **Multivariable analysis** | | |
| --- | --- | --- | --- | --- | --- | --- |
|  | **Hazard ratio** | **95%CI** | **p-value** | **Hazard ratio** | **95%CI** | **p-value** |
| Age (per 10 years increased) | 1.74 | 1.21-2.51 | 0.003 | 1.61 | 1.05-2.48 | 0.03 |
| Female | 1.58 | 0.64-3.88 | 0.32 | - | - | - |
| Diabetes mellitus | 1.48 | 0.58-3.77 | 0.42 | - | - | - |
| BMI (per 5 kg/m^2^ increased) | 1.40 | 0.95-1.06 | 0.09 | 1.30 | 0.85-1.99 | 0.23 |
| Living donor transplantation | 0.29 | 0.09-0.98 | 0.046 | 0.37 | 0.11-1.32 | 0.13 |
| HLA mismatch (per 1 mismatch increased) | 1.10 | 0.89-1.36 | 0.36 | - | - | - |
| CNI with MPA regimen (compared with other regimens) | 1.15 | 0.39-3.39 | 0.80 | - | - | - |
| Prednisolone-containing regimen | 1.02 | 0.40-2.62 | 0.96 | - | - | - |
| Serum creatinine (per 1mg/dL increased) | 1.05 | 0.92-1.21 | 0.45 | - | - | - |
| Time after transplantation (per 5 years increased) | 1.34 | 0.97-1.84 | 0.08 | 1.14 | 0.78-1.65 | 0.50 |
| One dose of vaccine received (compared with no vaccination) | 0.10 | 0.03-0.32 | <0.001 | 0.12 | 0.03-0.41 | 0.001 |
| Two doses of vaccine received (compared with no vaccination) | 0.09 | 0.03-0.27 | <0.001 | 0.11 | 0.03-0.34 | <0.001 |
| At least three doses of vaccine received (compared with no vaccination) | 0.06 | 0.02-0.22 | <0.001 | 0.06 | 0.01-0.23 | <0.001 |

BMI; body mass index, CNI; calcineurin inhibitor, HLA; human leukocyte antigen, MPA; mycophenolic acid

**Supplementary Table S2**: Univariable and multivariable Cox proportional hazards regression for pneumonia in kidney transplant recipients with COVID-19 infection.

| **Variables** | **Univariable analysis** | | | **Multivariable analysis** | | |
| --- | --- | --- | --- | --- | --- | --- |
|  | **Hazard ratio** | **95%CI** | **p-value** | **Hazard ratio** | **95%CI** | **p-value** |
| Age (per 10 years increased) | 1.38 | 1.14-1.67 | 0.001 | 1.32 | 1.05-1.64 | 0.016 |
| Female | 1.19 | 0.75-1.88 | 0.46 | - | - | - |
| Diabetes mellitus | 1.11 | 0.65-1.91 | 0.69 | - | - | - |
| BMI (per 5 kg/m^2^ increased) | 1.29 | 1.04-1.60 | 0.02 | 1.22 | 0.98-1.52 | 0.08 |
| Living donor transplantation | 0.21 | 0.10-0.43 | <0.001 | 0.27 | 0.13-0.58 | 0.001 |
| HLA mismatch (per 1 mismatch increased) | 1.07 | 0.96-1.20 | 0.21 | - | - | - |
| CNI with MPA regimen (compared with other regimens) | 0.80 | 0.47-1.33 | 0.39 | - | - | - |
| Prednisolone-containing regimen | 0.51 | 0.32-0.79 | 0.003 | 0.61 | 0.38-0.98 | 0.041 |
| Serum creatinine (per 1mg/dL increased) | 0.99 | 0.90-1.09 | 0.80 | - | - | - |
| Time after transplantation (per 5 years increased) | 1.20 | 1.00-1.44 | 0.05 | 1.09 | 0.89-1.34 | 0.39 |
| One dose of vaccine received (compared with no vaccination) | 0.21 | 0.12-0.38 | <0.001 | 0.26 | 0.14-0.47 | <0.001 |
| Two doses of vaccine received (compared with no vaccination) | 0.11 | 0.06-0.20 | <0.001 | 0.12 | 0.07-0.23 | <0.001 |
| At least three doses of vaccine received (compared with no vaccination) | 0.05 | 0.03-0.12 | <0.001 | 0.05 | 0.02-0.12 | <0.001 |

BMI; body mass index, CNI; calcineurin inhibitor, HLA; human leukocyte antigen, MPA; mycophenolic acid

**Supplementary Table S3**: Univariable and multivariable Cox proportional hazards regression for superimposed bacterial infection in kidney transplant recipients with COVID-19 infection.

| **Variables** | **Univariable analysis** | | | **Multivariable analysis** | | |
| --- | --- | --- | --- | --- | --- | --- |
|  | **Hazard ratio** | **95%CI** | **p-value** | **Hazard ratio** | **95%CI** | **p-value** |
| Age (per 10 years increased) | 1.42 | 1.07-1.88 | 0.016 | 1.43 | 1.06-1.91 | 0.017 |
| Female | 0.72 | 0.38-1.39 | 0.33 | - | - | - |
| Diabetes mellitus | 1.52 | 0.73-3.16 | 0.26 | - | - | - |
| BMI (per 5 kg/m^2^ increased) | 1.37 | 1.01-1.87 | 0.04 | 1.37 | 1.00-1.89 | 0.05 |
| Living donor transplantation | 0.36 | 0.15-0.87 | 0.02 | 0.47 | 0.19-1.14 | 0.10 |
| HLA mismatch (per 1 mismatch increased) | 1.03 | 0.87-1.21 | 0.74 | - | - | - |
| CNI with MPA regimen (compared with other regimens) | 1.56 | 0.61-4.01 | 0.36 | - | - | - |
| Prednisolone-containing regimen | 0.68 | 0.34-1.34 | 0.26 | - | - | - |
| Serum creatinine (per 1mg/dL increased) | 1.00 | 0.87-1.14 | 0.96 | - | - | - |
| Time after transplantation (per 5 years increased) | 1.15 | 0.88-1.52 | 0.31 | - | - | - |
| One dose of vaccine received (compared with no vaccination) | 0.17 | 0.06-0.45 | <0.001 | 0.19 | 0.07-0.53 | 0.001 |
| Two doses of vaccine received (compared with no vaccination) | 0.17 | 0.07-0.39 | <0.001 | 0.17 | 0.07-0.41 | <0.001 |
| At least three doses of vaccine received (compared with no vaccination) | 0.05 | 0.02-0.16 | <0.001 | 0.04 | 0.01-0.13 | <0.001 |

BMI; body mass index, CNI; calcineurin inhibitor, HLA; human leukocyte antigen, MPA; mycophenolic acid
